# Smoking Cessation With 20 Hz Repetitive Transcranial Magnetic Stimulation (rTMS) Applied to Two Brain Regions: A Pilot Study

**DOI:** 10.3389/fnhum.2018.00344

**Published:** 2018-09-24

**Authors:** Da Chang, Jian Zhang, Wei Peng, Zhuowen Shen, Xin Gao, Youhong Du, Qiu Ge, Donghui Song, Yuanqi Shang, Ze Wang

**Affiliations:** ^1^Department of Psychology, Center for Cognition and Brain Disorders, Hangzhou Normal University, Hangzhou, China; ^2^Department of Radiology, Lewis Katz School of Medicine, Temple University, Philadelphia, PA, United States

**Keywords:** smoking cessation, repetitive transcranial magnetic stimulation, brain entropy, cerebral blood flow, left dorso-lateral prefrontal cortex, superior medial frontal cortex

## Abstract

Chronic smoking impairs brain functions in the prefrontal cortex and the projecting meso-cortical limbic system. The purpose of this pilot study is to examine whether modulating the frontal brain activity using high-frequency repetitive transcranial magnetic stimulation (rTMS) can improve smoking cessation and to explore the changing pattern of the brain activity after treatment. Fourteen treatment-seeking smokers were offered a program involving 10 days of rTMS treatment with a follow-up for another 25 days. A frequency of 20 Hz rTMS was sequentially applied on the left dorso-lateral prefrontal cortex (DLPFC) and the superior medial frontal cortex (SMFC). The carbon monoxide (CO) level, withdrawal, craving scales, and neuroimaging data were collected. Ten smokers completed the entire treatment program, and 90% of them did not smoke during the 25-day follow-up time. A significant smoking craving reduction and resting brain activity reduction measured by the cerebral blood flow (CBF) and brain entropy (BEN) were observed after 10 days of 20 Hz rTMS treatments compared to the baseline. Although limited by sample size, these pilot findings definitely showed a high potential of multiple-target high-frequency rTMS in smoking cessation and the utility of fMRI for objectively assessing the treatment effects.

## Introduction

Cigarette smoking is a leading cause of preventable disease and premature death (Benowitz, 2010). Long-term smoking cessation, however, is known to be difficult for most smokers because of nicotine dependence (Shiffman et al., 2008; Hughes et al., 2011; Stead et al., 2012). Nicotine affects the brain by binding to the endogenous nicotinic acetylcholine receptors (nAchRs) (Ries et al., 2014), and subsequently disturbs the various brain functions regulated by the nAchRs. Chronical exposure to nicotine will upregulate the nAchRs, especially the nicotine high affinitive α4β2 subtype (Dani et al., 2001; Placzek and Dani, 2008) which is more abundant on dopaminergic neurons in the ventral tegmental area (VTA) and the projecting areas including the ventral striatum, medial orbito-frontal cortex (MOFC), anterior cingulate cortex (ACC), insula, amygdala, and dorsal prefrontal cortex (Brody, 2006; Ries et al., 2014), inducing neuroadaptation associated with abnormal dopaminergic activity in those areas (Hyman and Malenka, 2001; Robinson and Berridge, 2003; Volkow et al., 2003; Everitt et al., 2008; Kalivas and O’Brien, 2008). Due to the chronic nAchRs upregulation, abstinence from nicotine will dramatically increase the availability of unbound α4β2 nAchRs, which in turn leads to smoking craving (Staley et al., 2006) and the subsequent smoking relapse. Due to these neurobiological changes caused by chronic nicotine binding, treating smoking has proven to be highly difficult. The currently available effective methods, including medicines such as varenicline, bupropion, and nicotine replacement and psychotherapy, can still only provide around 25% abstinence rates at 6 months after the initial treatment (Hughes et al., 2011; Zwar et al., 2014).

At the macroscopic brain circuit level, the neurobiological effects of nicotine dependence converge to brain function disturbances. A potentially effective approach to treat nicotine dependence and smoking is to then revert the altered neural activity in the smokers’ brain. Transcranial magnetic stimulation (TMS) is a non-invasive neuromodulational tool (Hallett, 2000) that can be applied for the treatment of chronic smoking because of the long-lasting neuromodulational effects on repetitive application of TMS (the so-called rTMS) (Thickbroom, 2007; Ziemann et al., 2008). TMS works through a varying magnetic field created by frequently charging and discharging a capacitor. The magnetic field can penetrate the scalp losslessly and create a varying electrical field in the superficial cerebral cortex, which subsequently modulates the postsynaptic potentials of neurons in the cortex (Trojak et al., 1900; Fregni and Pascualleone, 2007; Rossini and Rossi, 2007; Slotema et al., 2010; Machado et al., 2013; Lan et al., 2016). By controlling the frequency of the varying magnetic field, the TMS can exert either excitatory or inhibitory effects on the target site and remote brain areas (Hallett, 2007). High frequency rTMS (≥5 Hz) often causes excitatory effects and low-frequency rTMS (≤1 Hz) is often cited for its inhibitory effects (Pascual-Leone et al., 1994; Chen et al., 1997). The rTMS has been assessed in chronic smoking in several studies. In Eichhammer (2003), reported that 20-Hz rTMS on the left DLPFC significantly reduced the number of cigarettes smoked during an *ad libitum* smoking period but without significant change in cigarette craving. Several subsequent studies found that a high frequency rTMS over the DLPFC reduced cue-induced craving in nicotine and cocaine/methamphetamine dependence (Camprodon et al., 2007; Politi et al., 2008; Li et al., 2013a; Dinur-Klein et al., 2014; Pripfl et al., 2014; Shen et al., 2016), while low frequency rTMS over the left DLPFC increased the craving for the drug (Li et al., 2013b), which was inconsistent with the findings of a later study where a low frequency rTMS over the left DLPFC was found to decrease drug craving (Flores-Leal et al., 2015). In Dinur-Klein et al. (2014) performed a large size (*n* = 77) rTMS-based cessation study by applying high frequency, low frequency, or sham stimulation for 13 days and then following up for 6 months. They reported a significant cigarette consumption reduction and nicotine dependence reduction on applying high- not low-frequency rTMS. Cigarette consumption reduction was further enhanced by combining the rTMS with smoking cue exposure. The 6 months abstinence rate was estimated to be 33%. Although very promising, their study was based on a customized deep stimulation coil, also called H coil, which has very limited availability due to the high cost and is nearly impossible to be applied to any other target brain site once the coil is designed. By contrast, the commonly used figure-of-eight-shaped coils can be readily applied to any superficial brain area and is available for any TMS system.

The purpose of this pilot study was to add to the early stage rTMS-based smoking cessation literature with new evidence. Two brain targets were chosen rather than one: the left DLPFC and the SMFC. The DLPFC is involved in inhibition control, which is known to be impaired in substance dependence. Exciting the DLPFC should then have the potential of enhancing the resistance capability for smoking. The SMFC has been shown to be activated while resisting drug craving (Hartwell et al., 2011) and is coupled with the orbitofrontal cortex, which is involved with impulsivity and is often implicated in drug-craving including smoking craving (Janes et al., 2014). Stimulating the SMFC should then provide a way to reduce smoking craving. Stimulating both the left DLPFC and the SMFC may increase the capability of inhibiting smoking and reducing the urge of smoking at the same time, leading to a more successful smoking cessation. To test this main hypothesis, both behavioral and neuroimaging data were collected to measure the treatment outcome. We previously showed that the chronic smokers had a more irregular resting state brain activity (Li et al., 2016), where the brain activity irregularity was measured by brain entropy (BEN). So one aim of this study was to test whether the rTMS can reduce BEN (Wang et al., 2014) in chronic smokers. Cerebral blood flow (CBF) is tightly coupled to the regional brain function and has been implicated in nicotine dependence (Franklin et al., 2006, 2011; Wang et al., 2007). In this study, we also collected the CBF data and aimed to test whether the rTMS treatment will alter the baseline CBF in smokers.

## Materials and Methods

### Participants

This study was conducted at the Center for Cognition and Brain Disorders (CCBD) in the Affiliated Hospital of Hangzhou Normal University. All procedures were approved by the CCBD Institutional Review Board and adhered to the Declaration of Helsinki. Signed written consent forms were collected before any procedure. Fourteen treatment-seeking smokers (13 males/1 female, age: 43 ± 14.5 years) who smoked for more than 5 years (and more than 10 cigarettes each day) participated in the study. Subjects were excluded if they suffered from diabetes, alcoholism, and psychiatric disorders.

### Design

**FIGURE 1:**
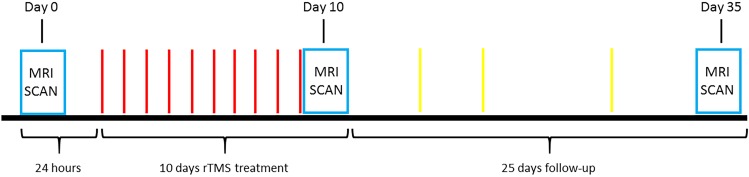
The paradigm of the entire experiment. On Day 0, each smoker participated in an MRI scan session to collect the structural imagine, resting state fMRI, and ASL data. With a 24-h abstinence from smoking after the first MRI scan the 10 days treatment began. A 20 Hz rTMS was applied on the left dorso-lateral prefrontal cortex and the superior medial prefrontal cortex, sequentially. At each daily rTMS treatment session, each site received 1000 pulses with a magnitude adjusted to 90% of the resting motor threshold. The CO level, withdrawal, and craving scales were collected every day. After the last treatment (Day 10), the smokers had the MRI taken that was same as Day 0. In the following 25 days the CO level, withdrawal, and craving scales were collected randomly. Again, at the end of F25 (Day 35), the MRI data were collected.

**Figure 1** illustrates the experimental paradigm used in this study. A total of 14 treatment-seeking smokers were offered a program involving 10 days rTMS treatment (T10) and 25 days follow-up (F25) after TMS and MRI safety screening. Subject exclusion criteria included: (1) metal in the brain or skull; (2) having pacemaker; (3) having medical implants such as intracardial lines or central venous catheter; (4) suffering from stroke, brain tumor or other brain trauma; (5) diagnosis of seizure or epilepsy; (6) undergone neurosurgery; (7) pregnancy; (8) having magnetic or electric implants in the body; (9) suffering from migraine; and (10) under medications. The rTMS treatment was started after a 24-h abstinence from smoking. A 20 Hz rTMS was applied on the left DLPFC and the SMFC. During each daily rTMS treatment session, 1000 TMS pulses with a magnitude adjusted to 90% of the resting motor threshold were applied to each of the two target sites, sequentially (LDLPFC followed by SMFC). Withdrawal, and craving scales were collected daily during T10 and several times on a random basis during F25 and at the end of F25, using the Minnesota Nicotine Withdrawal Scale (MNWS) (Cappelleri et al., 2005), together with the carbonmonoxide (CO) level. The CO level was used to monitor whether the subjects had smoked during the experiment. The research assistant was only explicitly asked to notice whether the subjects smoked based on a threshold value of CO < 7ppm, and the actual CO values were not documented for most of the subjects. Neuroimaging data were collected at the baseline (24 h before the first treatment), after T10 and F25 (**Figure 1**).

### rTMS Parameters

The rTMS was performed using a Magstim TMS machine and a figure-of-eight coil (Magstim Inc., Sheffield, United Kingdom). The left DLPFC rTMS target was set to be the place with a coordinate of (x,y,z) = (-40, 26, 37) mm in the Montreal Neurological Institute (MNI) standard brain space and mapped to each individual subject’s scalp using the BrainSight navigation system (Rogue Research Inc.). The SMFC rTMS target was located at one thumb width inferior to the midline of the left and right DLPFC according to the guidance of the probe placing rule in the electroencephalogram experiment. The coil was placed at an angle to the skull so that the magnetic field can act vertically on the cortex.

The rTMS was applied following the safety guidance provided by the International Workshop on the Safety of Repetitive Transcranial Magnetic Stimulator (Wassermann, 1998). For each site, the rTMS was administered in 20 successive pulse blocks interleaved with 28 s quitting time. Each block consisted of 50 pulses with 20 pulses per second (20 Hz) for 2.5 s. The magnitude of the pulse was set to be 90% of the resting motor threshold. The threshold was determined using the cycles of 10 single TMS pulses applied to the primary motor cortex. Beginning from low, the amplitude of the single pulse was gradually increased for each successive cycle until the thumb involuntarily moved five times within the same stimulation cycle. The amplitude of the final cycle was then used as the threshold.

### Imaging Parameters

Imaging experiments were performed on a 3.0T whole-body GE 750 MR scanner (GE, Milwaukee, WI, United States), using a standard 8-channel receive array. Structural images were acquired using a T1-weighted inversion prepared 3D spoiled gradient echo (IR-SPGR) sequence with FOV = 256 × 256 mm^2^, inversion time = 450 ms, TR/TE = 7.2/2.1 ms, matrix = 256 × 256, and 176 sagittal slices with slice thickness = 1 mm. The resting-state fMRI (rsfMRI) data, i.e., the blood oxygen level-dependent (BOLD) signal, were acquired with a T2^∗^ weighted gradient-echo echo-planar imaging (EPI) sequence with the following parameters: matrix = 64 × 64, voxel size = 3 × 3 × 3 mm^3^, and TR/TE = 2000/30 ms. A total of 37 axial slices were obtained in an interleaved order to cover the entire cerebrum and cerebellum from bottom to top. Arterial spin labeling (ASL) perfusion MRI was acquired with a GE product fast spin echo background suppressed 3D pseudo continuous ASL (pCASL) sequence with the following parameters: TR/TE = 4690/10.9 ms, 8 shots each with a spiral readout covering 512 k-space points, 40 axial slices, voxel size = 3.389 × 3.389 × 3.4 mm^3^, number of repeat scan (NEX) = 3, labeling duration = 1500 ms, post-labeling delay = 1525 ms, and the duration of the ASL scan was 4 min 32 s.

### Data Processing

The rsfMRI images were preprocessed using SPM12^1^ involving the following steps: (1) discarding the first 6 images to allow the signal to reach stead state; (2) the origins of both structural and raw rsfMRI images were set to be the center of the image matrix; (3) slice-timing correction; (4) motion correction; (5) registering rsfMRI to the MNI standard brain space via each subject’s T1 weighted anatomical image; (6) temporal nuisance correction using simple regression with the residual head motions, white matter signal, and cerebrospinal fluid (CSF) signal as the co-variants; (7) linearly detrending and temporal band-pass filtering (0.01–0.08 Hz) to eliminate high-frequency noise and low-frequency drift; and (8) spatial smoothing with an isotropic Gaussian kernel, full-width-at-half-maximum (FWHM) = 6 mm. Each subject’s BEN map was calculated based on the preprocessed rsfMRI images with BENtbx^2^ using an approximate entropy measurement, sample entropy (SampEn) with the parameters recommended in Wang et al. (2014); individual BEN maps were spatially normalized into the MNI space using the non-linear transform obtained by registering the structural MR image into the MNI space using SPM12.

Arterial spin labeling CBF images were processed using the SPM12-based batch scripts provided in ASLtbx (Wang et al., 2008). The following steps were included: (1) the origins of structural image, M0 map, and ASL images were set to be the center of the image matrix; (2) ASL CBF maps were calculated from the delta M and M0 maps and registered to the structural MR image for each subject; (3) CBF images were spatially normalized into the MNI using the similar procedure as mentioned above; and (4) CBF images were smoothed with an isotropic Gaussian filter with an FWHM = 6 mm.

Paired *t*-tests were performed on the preprocessed BEN maps and CBF maps to characterize the rTMS induced BEN and CBF changes, respectively. The statistical significance was defined by *p* < = 0.01 at the voxel level and a cluster size > = 270 [alpha < 0.5, corrected for multiple comparison using the Monte Carlo simulation-based correction approach provided in AFNI (the AlphaSim tool^3^)].

## Results

**FIGURE 2:**
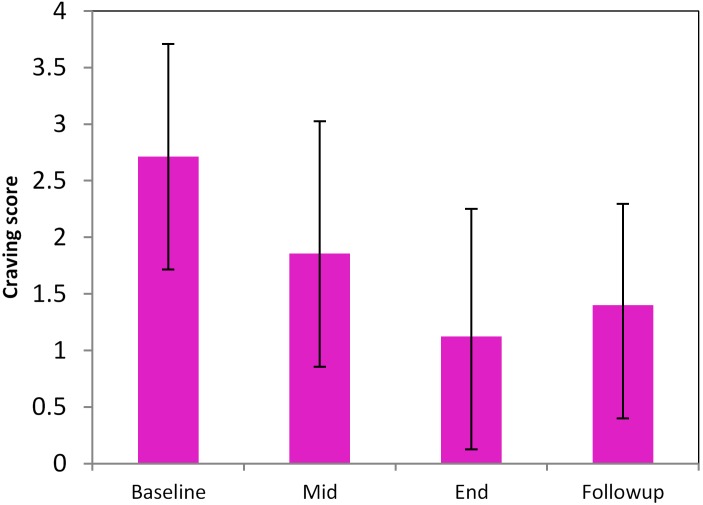
Longitudinal changes of smoking craving during the pilot high-frequency rTMS-based smoking cessation therapy. Baseline, Mid, End, and Follow-up mean the data acquired at the time before smoking cessation (smoking as usual), 5 days of rTMS treatment, 10 days of rTMS treatment (T10), 25-day follow-up (F25).

A total of 10 smokers completed the entire treatment program, of which 9 did not smoke during the entire period of 35 days. Complete MRI scans were only available for 8 subjects out of the 9 (all males, age: 47 ± 14.5). Withdrawal and craving at T10 and F25 were not significantly different; both were significantly reduced as compared to the baseline (**Figure 2**, *p* < 0.05).

**FIGURE 3:**
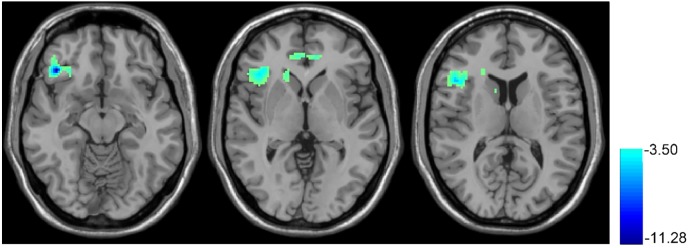
rTMS-induced BEN decrease. Paired *t*-test, when compared with the baseline condition (no rTMS), showed the 10 days rTMS treatment-induced CBF decrease in the right orbital part of the inferior frontal gyrus and right insula. In the thresholded *t*-map, blue means lower after rTMS treatment, *p* < 0.01, AlphaSim corrected (cluster size >270).

**Figure 3** shows the T10 (after rTMS) vs. baseline BEN comparison results. After 10 days of rTMS treatment, a significant BEN reduction was found in the right OFC, right anterior insula, right dorsal striatum, and ACC.

**FIGURE 4:**
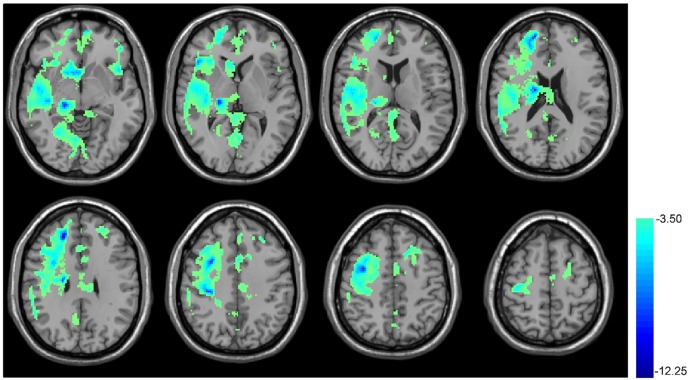
rTMS-induced CBF decrease in cerebral cortex. Paired *t*-test, when compared with the baseline condition (no rTMS), showed the 10 days rTMS treatment-induced CBF decrease in the right temporal lobe cortex, the big portion of the frontal lobe, precentral gyrus, caudate, hippocampus, cuneus, and thalamus as well as both sides of the insula. In the thresholded *t*-map, blue means lower after rTMS treatment, *p* < 0.01, AlphaSim corrected (cluster size >270).

**Figure 4** shows the results of CBF comparison between baseline and T10. The 10 days rTMS treatment induced a significant CBF reduction in a big portion of the cortical and sub-cortical areas in the right hemisphere including the right thalamus, right frontal and temporal cortex. The CBF reduction was also visible in the MOFC, ventral striatum, ACC, precuneus, left anterior insula, left PFC, and left DLPFC.

## Discussion and Conclusion

This pilot study showed that a 20 Hz rTMS sequentially applied to the left DLPFC and the SMFC for 10 days produced a high smoking cessation rate, which was confirmed by both the behavioral measures and the fMRI data: rTMS caused smoking cessation for the entire 25-day follow-up time, reduced craving for smoking, reduced BEN in the prefrontal cortex as well as insula, and decreased CBF in the right hemisphere. The promising high smoking cessation rate (90% of the 10 participants) and craving reduction were consistent with the previous studies (Camprodon et al., 2007; Politi et al., 2008; Li et al., 2013a; Dinur-Klein et al., 2014; Pripfl et al., 2014; Shen et al., 2016), suggesting that high-frequency rTMS is a promising tool to treat chronic smoking.

These encouraging preliminary results supported our hypothesis of stimulating the left DLPFC to increase the capability of inhibiting smoking and stimulating the SMFC to reduce the urge of smoking, thereby resulting in the high cessation rate. Brain imaging was adopted as an objective measure to monitor the treatment effects. Both the dynamic and static property of brain activity were assessed – the former was with BEN and the latter was with CBF.

BEN is a relatively new brain activity metrics (Song et al., 2018). Different from other more widely used activity measures, BEN indicates the irregularity of brain activity, which is related to the brain information processing capacity. The reduced BEN by rTMS found in the current study was supported by our previous smoking-as-usual study where we found that the chronic smokers had a higher BEN in a big portion of the cortex, with the greatest BEN increases located in the lateral striatum, prefrontal cortex, insula, middle and superior frontal cortex, and visual cortex (Li et al., 2016). Higher BEN in smokers might be a result of chronic psycho-stimulation by nicotine, similar to the increased BEN observed after taking psychedelic drugs (Carhart-Harris et al., 2014; Viol et al., 2017).

Smoking abstinence has been shown to cause regionally increased CBF (Boyajian and Otis, 2000; Zubieta et al., 2005; Wang et al., 2007), which was also related to abstinence-induced craving and withdrawal symptoms (Wang et al., 2007). The CBF reduction after 10 days of rTMS may then reflect a beneficiary brain effect of rTMS for smoking cessation, which was confirmed by the craving and withdrawal symptom reduction. Chronic smoking impairs the synthesis of nitric oxide and its regulatory role on CBF, resulting in a reduced CBF (Toda and Okamura, 2016). Accordingly, a full remission of nicotine dependence shall be accompanied with an increase of baseline CBF. This, however, could not be assessed in the current study due to the lack of MRI data acquired after the follow-up time.

As a preliminary study, there exist several limitations. The major limitation was the small sample size, which was mainly due to the difficulty of subject recruitment in Hangzhou. One possible reason is that smoking is still literally allowed in most public places. Another reason is the high work pressure and busy work schedule. Nevertheless, such a small sample size still yielded promising results, making it more valuable to perform a full validation study in the future. Related to the difficulty of subject recruitment, the second limitation was that there was no control group for controlling the placebo effects of rTMS. All the subjects declined to participate in the study once they heard that they might be assigned to a control group and receive a SHAM stimulation. We then revised the protocol to a non-blind one. Even with that modification, we could only recruit 14 treatment-seeking smokers within a period of 2.5 years. Another limitation was that there was no control for the two-site stimulation and the order of the sites. This makes it impossible to find whether two sites are better than only one target and whether the order matters.

To sum up, the high-frequency rTMS sequentially applied to the left DLPFC and SMFC may potentially be an effective tool for improving the smoking cessation rate; BEN and CBF provide valuable means to monitor or quantify the treatment effects.

## Author Contributions

All authors listed have made a substantial, direct and intellectual contribution to the work, and approved it for publication.

## Conflict of Interest Statement

The authors declare that the research was conducted in the absence of any commercial or financial relationships that could be construed as a potential conflict of interest.

## Footnotes

1 http://www.fil.ion.ucl.ac.uk/spm

2 https://cfn.upenn.edu/∼zewang/BENtbx.php

3 https://afni.nimh.nih.gov/
